# DC-SIGN (CD209)-mediated interactions between bacteria, lung cancer tissues, and macrophages promote cancer metastasis

**DOI:** 10.1186/s13027-025-00667-x

**Published:** 2025-06-21

**Authors:** Qiao Li, Nihal Hasan, Fei Zhao, Ying Xue, Sizhe Zhu, Yin Lv, Ling-yu Jiang, Kun Yang, Wenjin Li, Yingmiao Zhang, Yingxia He, Huahua Cai, Honghui Ding, John D. Klena, Andrey P. Anisimov, Shao-gang Wang, Hongxiang Chen, Chenglin Ye, Jingping Yuan, Tie Chen

**Affiliations:** 1 Tongji Hospital, Tongji Medical College, Huazhong University of Sciences and Technology, Wuhan, Hubei China; 2 Centers for Disease Control and Prevention, Atlanta, Georgia; 3 State Research Center for Applied Microbiology and Biotechnology, Obolensk, Russia; 4 Department of Urology, Tongji Hospital, Tongji Medical College, Huazhong University of Sciences and Technology, Wuhan 430000, Hubei, China; 5 Union Hospital, Tongji Medical College, Huazhong University of Sciences and Technology, Wuhan, 430060 People’s Republic of China; 6 Department of Pathology, Renmin Hospital of Wuhan University, 238 Jiefang-Road, Wuchang District, Wuhan, 430060 People’s Republic of China; 7 Department of Clinical Immunology, Tongji Hospital, Tongji Medical College, Huazhong University of Science and Technology, Wuhan, 430000 Hubei China

**Keywords:** Lung cancer, Metastasis, DC-SIGN, Gram-negative bacteria

## Abstract

One of the hallmarks of lung cancers is the earlier metastasis resulting from the dissemination of cancer cells. Although accumulating evidence suggests that bacterial infection may be involved in the development of the metastasis of lung cancer, few studies have explored the molecular mechanisms of bacterial infection in the dissemination of lung cancer cells. A series of studies have indicated that certain Gram-negative bacteria are able to hijack antigen-presenting cells (APCs) via interaction with DC-SIGN (CD209) receptors to facilitate the dissemination of pathogens, including viruses, bacteria, fungi, and parasites. Therefore, in the present work, it was hypothesized that bacterial infection may promote the dissemination of cancer cells via the utilization of a similar mechanism. It was first discovered that human lung cancer tissues contain a very high diversity of bacterial DNAs, indicating the co-existence of lung cancer tissues and microbial organisms. It was then found that lung cancer tissues express DC-SIGN, leading to binding with a Gram-negative bacterium, *Shigella sonnei*. Further, this bacterium was found to be able not only to induce the expression of DC-SIGN on macrophages but also to enhance the migration ability of lung cancer cells in vitro. The in vivo experiments supported these observations, showing that in wild-type (WT) mice, *Shigella sonnei* infection significantly increased tumor size, weight, and metastatic nodules compared to SIGNR1 knockout (KO) mice. These observations were associated with increasing DC-SIGN expression in WT mice. Finally, these results suggest that bacterial infections could play a significant role in promoting lung cancer progression and metastasis via DC-SIGN-mediated mechanisms.

## Introduction

Lung cancer continues to be a major global public health concern due to its very high morbidity and mortality rates [1–5]. It is divided into two histological types: non-small-cell lung cancer (NSCLC) and small-cell lung cancer (SCLC) [6, 7]. NSCLC accounts for over 85% of lung cancer cases and primarily includes adenocarcinoma (AC) and squamous cell carcinoma (SCC). Key risk factors for lung cancer include cigarette smoking, family history, genetic susceptibility, and chronic obstructive pulmonary disease [3, 8, 9]. Tumor metastasis, a hallmark of lung malignancies, is the primary cause of patient morbidity and mortality [10–12]. Despite extensive research, the molecular mechanisms driving lung cancer metastasis are still not fully understood.

The role of infection in cancer development is well-documented [13–18], with persistent lung infections notably associated with lung cancer [19]. Various studies have employed bacterial 16S ribosomal RNA sequencing to examine the microbiota of lung tissues [20–33]. Nejman et al. [32] detected tumor-type-specific intracellular bacteria in various human tumors, including lung cancer, with these bacteria predominantly found within tumor cells and immune cells, potentially being carried by immune cells from other body regions [32].

For metastasis to occur, tumor cells need to acquire various capabilities such as migration through the lymphatic and blood systems, secretion of enzymes that degrade the extracellular matrix, formation of a pre-metastatic niche, and alteration of the local tumor microenvironment (TME) [11, 34, 35]. Metastasis is closely related to chronic inflammation [36, 37]. While inflammation is an evolutionary strategy for host defense against pathogens, the interaction between immune cells and persistent bacterial infections can also trigger tumor metastasis [37]. Firstly, inflammation can alter the TME, with macrophages playing a pivotal role. These macrophages can adopt a tumor-promoting phenotype following infection with various bacteria, such as *Salmonella typhimurium* and *Chlamydia pneumoniae*, thereby enhancing cancer cell migration [38, 39]. Second, chronic inflammation and bacterial infection have been associated with epithelial-mesenchymal transition (EMT) activation, a critical process in lung cancer metastasis [40]. During EMT, tumor cells transition from epithelial characteristics to mesenchymal properties [41–43]. Microbial pathogen infection can induce EMT, enhancing tumor metastasis [40]. Third, bacterial infection can activate metastasis through the induction of matrix metalloproteinases (MMPs) [44]. MMP activation leads to the degradation of the tissue matrix, facilitating the migration of tumor cells, including lung cancer cells, across tissue barriers [45, 46]. Finally, tumor-associated macrophages (TAMs) also play a role in metastasis. For instance, a hypoxic tumor microenvironment in NSCLC can promote lung cancer cell metastasis by polarizing macrophages to acquire the DC-SIGN-expressing M2 phenotype [47–49].

Antigen-presenting cells (APCs) located along mucous membranes continuously monitor for bacteria entering from the gut lumen and respiratory tract [50, 51]. Phagocytosed bacteria can be transmitted through the lymphatic system to the reticuloendothelial system, triggering an active immune response. Under normal conditions, bacterial translocation, where intestinal bacteria pass through the intestinal wall, enter the bloodstream, and disseminate to other sites, rarely leads to bacteremia. However, certain clinical circumstances, such as immunosuppression, impaired mucosal integrity, or colitis, can increase the risk of severe disease caused by circulating bacteria [52, 53]. C-type lectin receptors play a crucial role as innate immune pattern recognition receptors (PRRs) on APCs such as macrophages, facilitating the interaction between host cells and pathogens [54–56]. Macrophages express high levels of C-type lectin receptors, such as Dendritic cell-specific intercellular adhesion molecule-3-grabbing non-integrin (DC-SIGN), which include one or more carbohydrate recognition domains (CRDs) capable of binding to pathogen-specific carbohydrate ligands [57–71]. Several studies have demonstrated that various Gram-negative bacteria, such as *Salmonella enterica serovar Typhimurium, Helicobacter pylori, Escherichia coli, Yersinia pseudotuberculosis, Neisseria gonorrhoeae, Yersinia pestis, Haemophilus ducreyi, P. mirabilis*, and *Shigella spp*., can bind to DC-SIGN receptor utilizing their LPS core [57–63, 66, 67, 70, 71]. This interaction enables Gram-negative bacteria to enhance their dissemination within the host [60–62, 70, 71]. Interestingly, DC-SIGN expression has been observed in various cancer cells, including NSCLC, colon cancer, and gastric cancer cells [47, 72], and has been associated with enhanced migration and metastatic potential. Zhang et al. found that higher DC-SIGN expression levels in lung cancer tissue samples were associated with the metastasis stage [47]. However, the underlying mechanism of DC-SIGN expression in lung cancer and its role in lung cancer carcinogenesis remain not fully understood.

*Helicobacter pylori* has been implicated in lung carcinogenesis [73–75]. It has been suggested that persistent bacterial infection in the gut can compromise the gut mucus layer, allowing bacteria to penetrate the intestinal layer and be transported to the lungs by immune cells [32]. However, the cultivation of this pathogen requires an anaerobic environment. Therefore, another Gram-negative bacterium, *Shigella sonnei* (*S. sonnei*) [76], was selected for this study. *Shigella* is an intracellular pathogen with subgroups including *S. sonnei, S. boydii, S. dysenteriae,* and *S. flexneri* [76, 77]. *Shigella* is known to cause bacillary dysentery and invade the colon and rectum mucosa. Although rare, this bacterium can enter the bloodstream [78, 79]. *Shigella* may enter the colorectal mucosa through microfold cells (M cells), which transport antigens to the subepithelial space where gut-associated lymphoid tissue and APCs such as macrophages are located. In the colon and rectum, APCs act as "Trojan horses," facilitating the crossing of the intestinal epithelial barrier [70]. The O-antigen-encoding genes in *S. sonnei* are located on the 210-kb virulence plasmid, which is unstable and easily lost during bacterial culture [70]. Previous studies have shown that when *Shigella* loses this large plasmid, it also loses the expression of O-antigen and transforms into a rough strain [70, 80–82]. The rough strain of *Shigella* can interact with macrophages through DC-SIGN receptors, thereby promoting host dissemination [70]. In addition to its role as an intestinal pathogen, clinical studies have documented *Shigella sonnei*-associated pneumonia, both with and without dysentery, in immunocompetent and immunocompromised individuals [83–87]. Furthermore, *S. sonnei* has been detected in respiratory specimens from lung cancer patients with pneumonia [88], highlighting its clinical relevance in lung cancer-related respiratory complications. Considering these characteristics of *S. sonnei*, this strain was used as the model strain in the present study.

This study aims to investigate whether Gram-negative bacterial infection promotes lung cancer progression and metastasis via DC-SIGN-mediated mechanisms. Understanding this interaction could provide new insights for developing novel therapeutic strategies to treat lung cancer.

## Materials and methods

### Ethics statement

All animal procedures and human experiments were conducted in strict accordance with the Institutional Animal Care and Use Committees (IACUCs) and Institutional Review Board (IRB) of Tongji Hospital, Tongji Medical College, China. All experimental procedures were specifically approved for this study by the Medical Ethics Committee of Tongji Hospital (IRB ID: TJ-A20141220 for animal experiments and TJC20140113 for human experiments) and were carried out in accordance with institutional guidelines. Lung cancer tissues were derived from lung cancer patients recruited at Renmin Hospital of Wuhan University. All volunteers involved in the experiment gave signed informed consent.

### Bacterial strains

*Escherichia coli K-12* strain CS180 is a rough LPS *E. coli* strain. CS1861 is the derivative of CS180, modified with the pSS37 plasmid, which carries the complete set of genes required for *Shigella dysenteriae* 1 O-antigen expression [89, 90].

*Yersinia pseudotuberculosis* Y1, a serotype O:1a strain, is missing the virulence plasmid (pYV) [91–93].

*P. mirabilis* is a clinical isolate lacking O-antigen expression. *P. mirabilis* PAY100.1 is *P. mirabilis* modified with the pAY100.1 plasmid that contains all the genes required for the expression of the O-antigen of *Y. enterocolitica* serotype O: 3 [94, 95].

*S. sonnei* is a clinical isolate lacking O-antigen expression. *S. sonnei*-*O*^+^ is the *S. sonnei* rough strain transformed with pSS37, a plasmid that carries all the genes required for the expression of *S. dysenteriae* 1 O-antigen (smooth) [70].

### Cell lines

RAW264.7 is a murine macrophage cell line, Lewis lung carcinoma (LLC) is a mouse lung cancer cell line, and PC-9 is a human NSCLC cell line. All the above cells were cultured in RPMI medium supplemented with 10% fetal bovine serum (FBS), streptomycin (100 μg/ml), and penicillin (100 U/ml), and then incubated at 37 °C and 5% CO_2_.

### Mice

C57BL/6 wild-type (WT) mice used in this study were obtained from the Huazhong University of Science and Technology Animal Center. SIGNR1 knockout (SIGNR1 − / −) (KO) mice, which shared the same genetic background as the WT mice but lacked SIGNR1 (SIGNR1-/-), were supplied by the Consortium for Functional Glycomics (http://www.functionalglycomics.org). The age and weight of the WT and SIGNR1 − / − mice utilized were matched. All mice were housed and fed under standard conditions for more than two weeks before the experiment. Moreover, all mice were treated following the Animal Care Committee guidelines of Tongji Hospital.

### Reagents

Mouse anti-human DC-SIGN was purchased from Santa Cruz Biotechnology (Delaware Ave, CA, USA). Mouse anti-SIGNR1 conjugated to allophycocyanin (APC) was purchased from eBioscience, Shanghai, China. Mouse anti-SIGNR1 was purchased from Bioss, China. A mouse IL-10 ELISA kit (EMC005) was purchased from NeoBioscience, China. Interleukin 4 (IL-4) was purchased from Peprotech, USA. For cell culture, RPMI medium was obtained from (gibco, China), FBS was obtained from Zhejiang Tianhang Biotechnologies, and Tripsin-EDTA solution and antibiotics were obtained from Biosharp, China. Triton X-100 was purchased from Biosharp, China. Trybtone, yeast extract, NaCl, and agar were purchased from Biosharp, China. For phosphate-buffered saline (PBS), all reagents were purchased from Biosharp, China.

### Bacterial tissue adhesion assay

Fresh lung cancer tissues derived from patients were cut into pieces with a final volume of 2 mm^3^. The tissue pieces were resuspended in RPMI with 2% FBS and placed in individual wells of a 24-well plate at a volume of 1 ml/well, in triplicate. Next, 50 μl bacterial suspensions (OD600nm = 0.2) of *Yersinia pseudotuberculosis, Proteus mirabilis,* and *Shigella sonnei* were added to each well. As controls, *E. coli* K-12 strain CS180 and CS1861 were also included. The bacterial suspensions and tissues were incubated for 2 h at 37 °C in 5% CO2. After incubation, the plate was gently washed with PBS three times to remove any unattached bacteria.

To evaluate bacterial adhesion, the tissues were collected and homogenized in 1% Triton X-100 using a tissue grinder. The resulting suspension was serially diluted in PBS and plated onto Luria–Bertani (LB) agar plates. The number of colony-forming units (CFUs) was determined, and bacterial adhesion levels to tumor samples were calculated by counting the CFUs recovered from the lysed tissue samples.

In the inhibition experiment, 5 μg/ml of anti-hDC-SIGN antibody and 500 μg/ml of mannan were used to block the receptor-bacterial interaction. These inhibitors were added to the wells 20 min before the addition of the bacterial suspensions to evaluate their effect on bacterial adhesion. The concentrations used in the experiments were determined according to protocols established in previous studies [58, 71].

### Histopathological and immunohistochemical analysis

After the mice were sacrificed at the designated time point, the mouse lung tissues were collected in a sterile environment. The tissues were fixed with 4% paraformaldehyde (PFA), embedded in paraffin, and sectioned at the largest cross-sectional area. Hematoxylin, eosin, and Masson’s trichrome were used for staining. The histopathology of lung carcinoma was then observed.

Human lung cancer tissues, paired remote normal tissues, and normal lung tissues were obtained from the Renmin Hospital of Wuhan University. The mouse lung cancer tissues were harvested from C57BL/6 J mice. Immunohistochemical analysis of the human lung cancer tissues and paired remote normal tissues was performed using an anti-human DC-SIGN antibody. The anti-mouse SIGNR1 antibody was used for immunohistochemical analysis of the mouse lung cancer tissues. The expression of human or mouse SIGNR1 was then analyzed.

The human lung cancer tissues, paired remote normal tissues, normal lung tissues, and mouse lung cancer tissues were cut into sections of 2–3 mm^3^ and embedded in paraffin. The antigen retrieval was done by heating with a citrate buffer (pH 6.0) at 100 °C for 8 min. Next, the slides were sealed with goat serum for 1 h and incubated with a primary antibody against human or mouse DC-SIGN for 1 h. After rinsing in PBS three times, the corresponding secondary antibody was added and incubated for 2 h. Finally, DAB chromogen solution (brown immunoperoxidase 3,3'-diaminobenzidine) was added and the slides were observed under an ECLIPSE CI microscope (Nikon, Japan) and analyzed using ImageJ 1.50 software (National Institutes of Health, Bethesda, MA, USA).

### Lung cancer tissue bacterial profiling

Bacterial DNA was extracted from lung cancer tissue samples derived from patients, as previously described [32]. Polymerase chain reaction (PCR) amplification of the 16S ribosomal RNA (rRNA) genes was performed on the extracted bacterial DNA using the StepOnePlus™ Real-time PCR System (Thermo Fisher Scientific, Waltham, MA, USA). Sequencing of the 16S rRNA gene was then performed using third-generation Pacific Biosciences (PacBio) single-molecule real-time (SMRT) sequencing technology. 16S rRNA gene sequencing is commonly used to analyze the bacterial composition of different samples [96]. The 16S rRNA gene contains highly conserved sequences interspersed with nine hypervariable regions. An accurate bacterial profile was obtained by comparing the 16S rRNA gene sequences from the DNA extracted from the lung cancer samples. Long-read sequencing using SMRT technology can capture full-length 16S rRNA sequences and determine the microbiome of the sample at the genus and species level.

### Preparation of LLC supernatant-treated RAW264.7 conditioned medium

RAW264.7 cells were seeded in a 24-well plate at a concentration of 2 × 10^5^ cells/well. After overnight culturing, live or heat-killed gram-negative *S. sonnei* (100 °C, 50 min), 10 ng/ml IL-4, and 1 mL PC-9 supernatant, with or without heat-killed Gram-negative *S. sonnei,* were added to the cell layer. The medium in the plate was replaced with serum-free RPMI-1640 medium after 24 h of co-culture. After 48 h, the medium in the plate was collected, filtered using a 0.2-μm filter, and used as the conditioned medium (CM) after processing the RAW264.7 for subsequent experiments.

### Flow cytometry

The SIGNR1 expression on RAW264.7 cells was examined by flow cytometry. A total of 10^6^ RAW264.7 cells were washed in PBS and placed in FACS tubes. Then, 10 ml PBS were added to the cells, after which they were centrifuged at 1000 rpm for 6 min and washed twice. Next, 1 μl anti-SIGNR1 antibody (1:100 dilution; eBioscience, China) was added to the FACS tubes. The tube contents were then fully mixed and incubated at 4 °C for 20 min. After staining, the cells were washed in PBS, centrifuged at 1000 rpm for 6 min, and washed twice. SIGNR1 expression was examined on a FACSCanto flow cytometer (BD Biosciences) and analyzed using CellQuest Pro software (BD Biosciences).

### Transwell assay

Lung cancer cells (LLC and NSCLC cell lines) were cultured with RAW264.7 cells in a Transwell Boyden chamber (8-μm pores, BD Biosciences) and then seeded at a concentration of 1 × 10^5^ cells/100 μl of serum-free medium supplemented with 1% bovine serum albumin (BSA) into the inserts. RAW264.7 cells were seeded at a concentration of 1 × 10^5^ cells/500 μl of complete medium into the lower chamber. After co-culturing at 37 °C for 24 h, the migrant cells on the bottom of the filter were fixed with 4% PFA at 4 °C for 15 min and stained with Giemsa solution. An image was then recorded with a Light microscope.

The lung cancer cells were seeded at a concentration of 10^5^ cells/well for 48 h in a serum-deprived medium supplemented with 1% BSA in the upper chamber of a Transwell Boyden chamber (8-μm pores, BD Biosciences). The *S. sonnei-*infected, LLC supernatant–treated RAW264.7 CM, or complete medium was added to the lower chamber at a volume of 500 μl. After culturing for 48 h, the cells on top of the insert were removed using a cotton swab; and the cells on the bottom of the filter were fixed with 4% PFA at 4 °C for 15 min and stained with Giemsa solution. Images were recorded using a Light microscope.

### Wound-healing assay

A wound-healing assay was performed to assess cell migration by determining the ability of the cells to move into an acellular space. Approximately 5 × 10^5^ cells (the LLC and PC-9 cell lines) were added to the 6-well plate. After 24 h, the cell monolayer was wounded by scratching the surface on the 6-well plate as uniformly as possible using a 1 ml pipette tip. The cells were washed in PBS three times to remove the cell debris, and the *S. sonnei-*infected CM was added. The initial wounding and the movement of the cells in the scratched area were photographed using an Olympus CKX41 inverted microscope equipped with a digital imaging system at 0 and 24 h. The wound width of six random views was measured.

### Lung tumor metastasis model

The animal model experiment was carried out as follows:

Preparation of C57BL/6 J Mice: wild-type (WT) mice and knock-out (KO) mice were divided into four groups, with ten mice in each group. To initiate the experiment, *S. sonnei* bacteria (OD600 = 0.5, 30 μl/mouse) were administered nasally to 10 WT and 10 KO mice. Another set of mice with the same genetic background was used as controls (10 mice per group) and were nasally administered a normal saline solution (0.9% NaCl). After 6 h, all the mice were injected with LLC cells (1 × 10^7^ cells per 100 μl) through their tail veins. Tumor growth was monitored by tracking body weight every two days, and mice were euthanized if they lost more than 10% of their initial body weight or at the 24-day endpoint, whichever came first. Based on this, tumors were first measurable approximately 10–14 days after inoculation, when the weight loss criterion was met. Tumor metastases and burdens were evaluated by assessing total lung weights and sizes at the time of euthanasia:

- The lung weight was measured using a precision balance.
- Lung sizes were measured using a vernier caliper clamp and calculated using the formula:
  $$\left( {{\text{mm3}}} \right) \, = \, \left( {{\text{L }} \times {\text{ W2}}} \right) \, \times \, 0.{5}$$

Subsequently, histopathology analysis was conducted to examine the pathological characteristics in the lung cancer tissues of all mouse groups. Immunohistochemical analysis was performed to investigate the expression of SIGNR1 in the lung cancer tissues of the WT mice.

### Statistical analyses

All experiments were conducted in three independent replicates. Prism soft-ware, version 6 (Graph Pad, San Diego, CA, USA) and Origin (version 2019, OriginLab Corporation, Northampton, MA, USA) were used for analysis. A P-value of 0.05 (*P < 0.05, **P < 0.01, ***P < 0.001) was considered the threshold for statistically significant differences and calculated using the Student t-test for the univariate analysis of two sets of data and two-way analysis of variance (ANOVA) for multiple comparisons.

## Results

### Assessment of lung cancer tissue bacterial composition

**Fig. 1:**
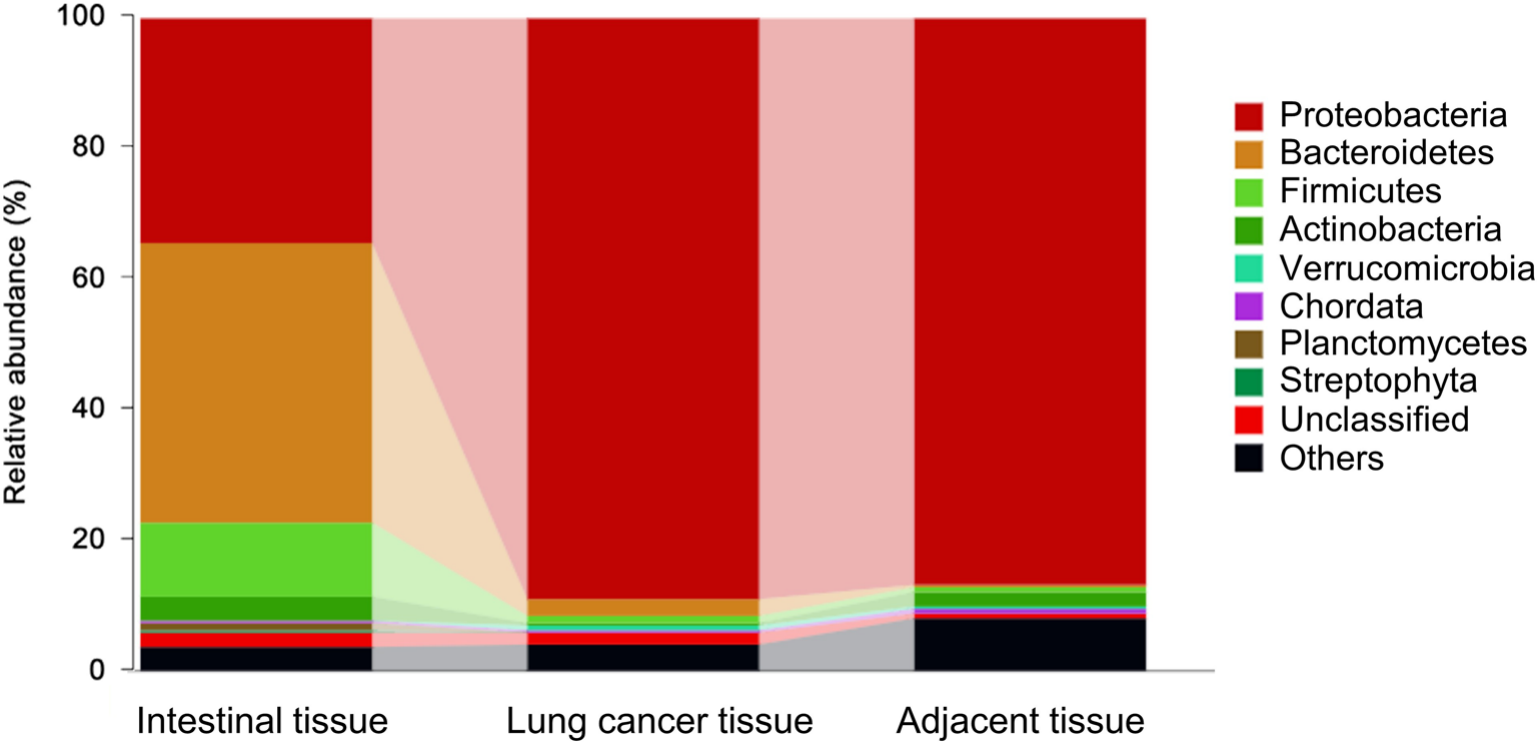
Microbiota determination in lung tissues. This figure displays differences in the abundance of microbiome members in various lung cancer tissue and adjacent normal tissue samples. The 7000 selected sequences of lung cancer tissue and adjacent normal tissue specimens were sequenced, and the bacterial OTUs of the lung cancer tissue and adjacent normal tissue specimens were ranked by abundance. The proportion of each out was determined by the column length

Profiling the bacterial composition of human lung cancer and the adjacent normal tissues provided a bacterial profile of the lung cancer tissue. High-throughput 16S rRNA sequencing was employed to analyze the lung microbiome, a widely utilized method in various tumor samples [26, 32, 97, 98]. Operational taxonomic unit (OTU)-level analyses were performed to identify differentially abundant OTUs between lung cancer tissues and adjacent normal tissues. As shown in Fig. 1**,** Proteobacteria was the predominant phylum in lung cancer and adjacent tissues. In addition, a slightly higher proportion of Bacteroidetes was observed in cancerous tissues compared to adjacent normal tissues. These findings indicate that human lung cancer tissues contain a diverse population of Gram-negative bacteria, which could potentially contribute to the mechanisms underlying lung cancer progression.

### Expression of human DC-SIGN in the lung cancer tissues

**Fig. 2:**
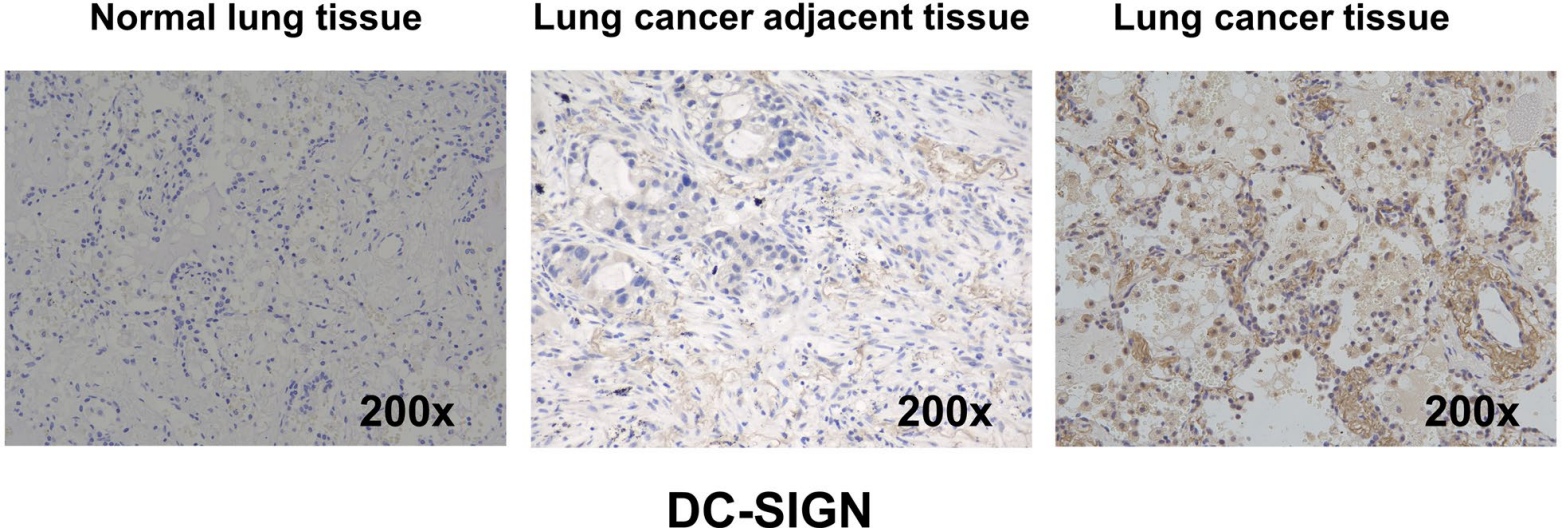
DC-SIGN expression in normal lung tissue, lung cancer adjacent tissue, and lung cancer tissue. DC-SIGN expression was examined in normal lung tissue, lung cancer tissue, and adjacent normal tissue. Immunostaining for DC-SIGN via the brown immunoperoxidase 3,3'-diaminobenzidine (DAB) technique was described in the materials and methods section. Left, DC-SIGN was detected in normal lung tissue. Middle, DC-SIGN was detected in lung cancer adjacent tissue. Right, DC-SIGN was detected in lung cancer tissue. Magnification, 200 ×

Accumulating evidence suggests that DC-SIGN may play a significant role in various aspects of carcinogenesis [39, 47]. The expression of C-type lectin receptors, such as DC-SIGN, has been reported in different types of tumors [47, 99, 100]. Immunohistochemical analysis was performed on samples from lung cancer patients using an anti-human DC-SIGN antibody to examine the expression of human DC-SIGN in lung cancer tissues. As shown in Fig. 2**,** DC-SIGN expression was minimal in normal human lung tissues and adjacent normal tissues near the lung cancer site. On the other hand, lung cancer tissues exhibited increased DC-SIGN expression compared to lung cancer tissues compared to normal lung tissue negative controls and adjacent normal lung tissues. These findings were consistent with results from previous studies. For example, Zhang et al. [47] showed that DC-SIGN was expressed in NSCLC tissues and that its expression level is positively correlated with the tumor's metastasis stage. These findings indicate a potential role for DC-SIGN in the pathogenesis and progression of lung cancer, supported by its heightened expression, specifically in lung cancer tissues.

### Gram-negative bacteria interact with human lung cancer tissues via DC-SIGN

Previous studies have shown that the core LPS of Gram-negative bacteria can bind to C-type lectin receptors, such as Langerin and DC-SIGN, facilitating bacterial dissemination within the host [57–60, 62, 63, 66–71]. Following the evaluation of DC-SIGN expression in lung cancer tissues, the investigation focused on the potential role of DC-SIGN in mediating interactions between lung cancer cells and Gram-negative bacteria. Consequently, the adherence abilities of *S. sonnei*, *P. mirabilis*, and *Yersinia pseudotuberculosis* to lung cancer tissues were examined. *E. coli* K-12 strains CS180 (rough strain) and CS1861 (smooth strain) were used as a control [57].

**Fig. 3:**
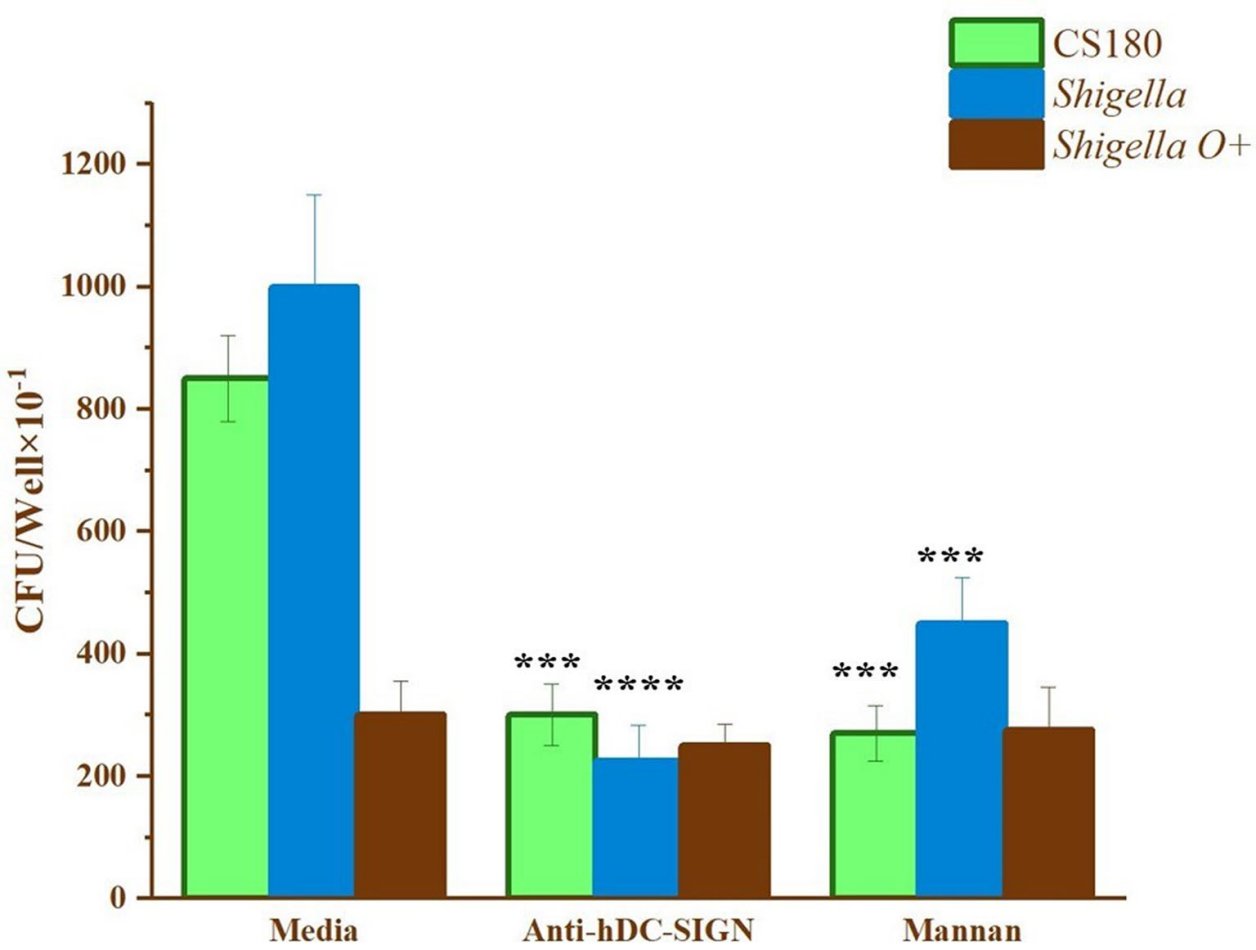
Gram-negative bacteria interact with human lung cancer tissues via DC-SIGN. The sets of bacteria, *E. coli* K-12 CS180, and CS1861 strains. **A** *Shigella* and *Shigella*-pAY100.1, **B** *P. mirabilis* and *P. mirabilis*-pAY100.1, and **C** *Yersinia pseudotuberculosis* cultured at 37 °C and 26 °C were respectively used in an adhesion assay to determine the rate of adherence to the lung tissue. The data presented were pooled from three independent experiments. *P < 0.05, **P < 0.005, ***P < 0.0001. *Shigella* O + : *Shigella*-pAY100.1; Pmi: *P. mirabilis*; Pmi O + : *P. mirabilis*-pAY100.1; Y1 37: *Yersinia pseudotuberculosis* cultured at 37 °C; Y1 26: *Yersinia pseudotuberculosis* cultured at 26 °C

Results demonstrated that *S. sonnei* interacted with human DC-SIGN, enhancing its adherence to lung cancer tissues. As shown in Fig. 3A, S*. sonnei* adhered to lung cancer tissues more effectively than *S. sonnei-O*^+^. Additionally, the interaction between *S. sonnei* and lung cancer tissues has been blocked by O-antigen expression, indicating that the binding of lung cancer tissues to *S. sonnei* is mediated by the interaction between human DC-SIGN and bacterial core LPS. Subsequently, further investigations were performed to explore the potential for similar interactions with other Gram-negative bacteria. Therefore, the binding ability of *P. mirabilis* with human lung cancer tissues was assessed, as shown in Fig. 3B. *P. mirabilis* adhered to lung cancer tissues much more effectively than *P. mirabilis*-pAY100.1, indicating that the *P. mirabilis*-lung cancer interaction is blocked by O-antigen expression. In short, these results demonstrate that *P. mirabilis* could interact with hDC-SIGN to enhance its adhesion to lung cancer tissues. The ability of *Yersinia pseudotuberculosis* to adhere to lung cancer tissues was also examined (Fig. 3C). During infection, *Y. pseudotuberculosis* has shown the ability to suppress the expression of its virulence factor, namely the O-antigen. This suppression occurs in bacteria cultivated at 37 °C [101–103], indicating that the LPS core may be exposed during in vivo growth. Considering the potential loss of O-antigen in *Y. pseudotuberculosis* cultured at 37 °C, the interaction between lung cancer tissues and *Y. pseudotuberculosis* grown at both 37 °C and 26 °C was investigated. As shown in Fig. 3C, the *Y. pseudotuberculosis* cultured at 37 °C adhered to lung cancer tissues more effectively than when grown at 26 °C. These results suggested that *Y. pseudotuberculosis* via their LPS core could interact with hDC-SIGN to enhance its adhesion to lung cancer tissues.

All the above results suggested that DC-SIGN^+^APCs, such as macrophages, can interact with Gram-negative bacteria at remote sites, such as the gut, and potentially transport these bacteria to the lungs to contribute to cancer progression. Furthermore, it is also plausible that lung cancer tissues may upregulate the presence of DC-SIGN^+^ cells, thereby facilitating the dissemination of bacteria from other body parts to the lung tissues.

### The interaction between *Shigella sonnei* and lung cancer tissues can be inhibited by anti-hDC-SIGN antibody and mannan

In the adherence assay, different species of Gram-negative bacteria were examined to investigate their interactions with lung cancer tissue via the DC-SIGN receptor. This diversity facilitated a comprehensive evaluation of binding affinity and receptor engagement among various pathogens. For subsequent experiments, *S. sonnei* was used as the bacterial model because *S. sonnei* causes shigellosis, a common intestinal disease, and does not require special growth conditions. The O-antigen in *S. sonnei* is easily lost during bacterial culture. Furthermore, its ability to invade APCs, such as mouse macrophages, through the DC-SIGN receptor and facilitate host dissemination [70] made it a particularly relevant model for our previous hypothesis.

**Fig. 4:**
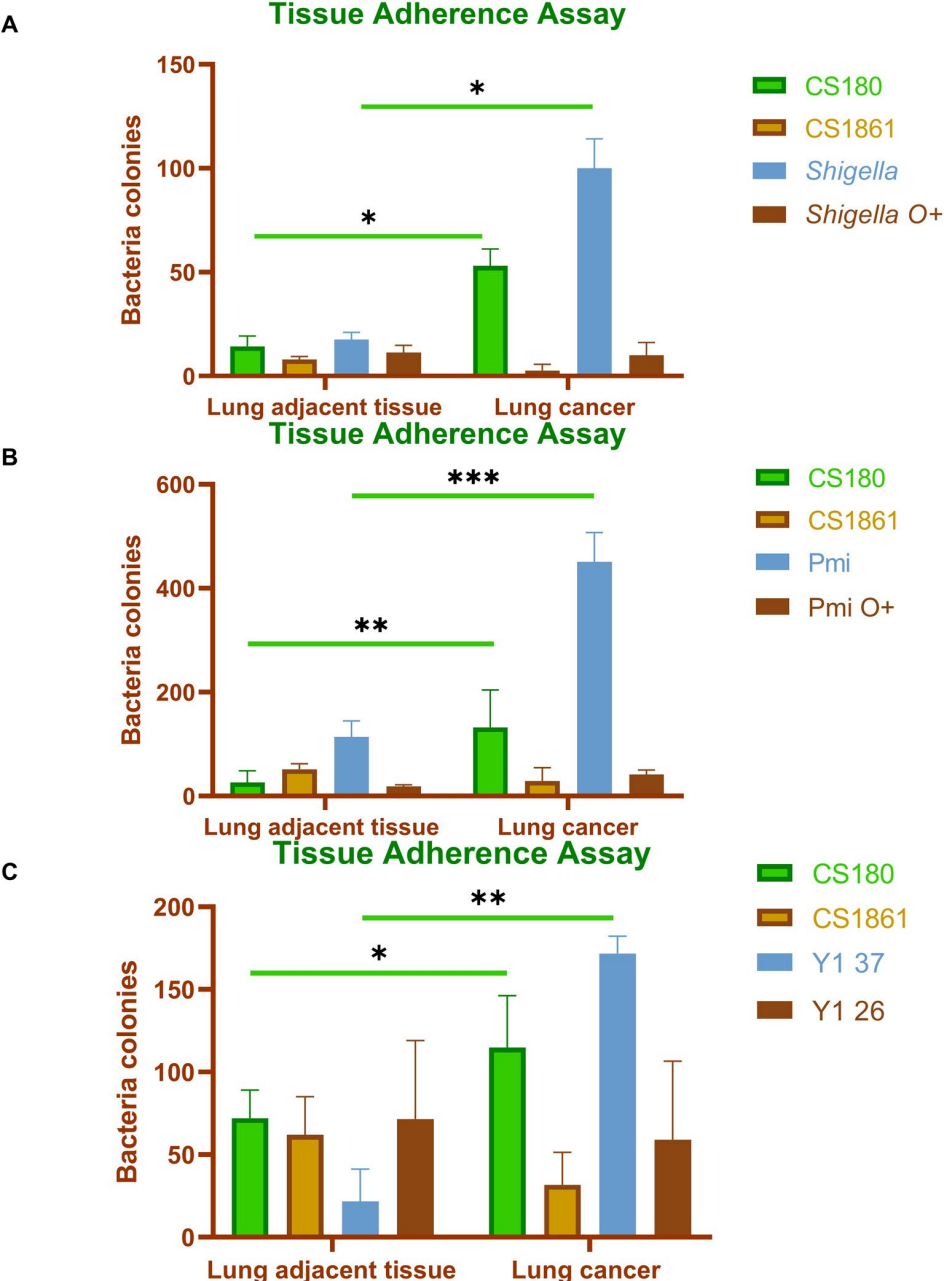
Inhibition of *Shigella sonnei* adherence to lung cancer Tissues by anti-hDC-SIGN Antibody and mannan. *Shigella sonnei* were incubated with lung cancer for 2 h, either with or without anti-hDC-SIGN and mannan. *E. coli* K12 CS180 served as the control strain. These results are based on data pooled from three separate experiments. ***P < 0.001, ****P < 0.0001

Inhibition assays were performed to determine whether the interaction between *S. sonnei* and lung cancer tissues is mediated by the DC-SIGN receptor. As shown in Fig. 4, bacterial adherence to lung cancer tissues was significantly reduced following treatment with both mannan and anti-hDC-SIGN antibodies. However, mannan was less effective than the anti-hDC-SIGN antibody in inhibiting bacterial adherence. These results suggest that the DC-SIGN receptor plays a critical role in mediating the interaction between *S. sonnei* and lung cancer tissues, with the potential involvement of other mannose-binding receptors. The significant reduction in adherence following anti-hDC-SIGN treatment further supports the critical role of the DC-SIGN receptor in this binding process.

### The expression of SIGNR1 was increased on macrophages following gram-negative bacterial infection

Previous studies have demonstrated that several Gram-negative bacteria, including *S. sonnei*, can interact with DC-SIGN expressed on APCs to facilitate their dissemination within the host [57–60, 62, 63, 65–71, 104–106]. However, it remains unclear whether Gram-negative bacteria can induce the upregulation of DC-SIGN in macrophages, thereby promoting the metastasis of lung cancer cells. Therefore, we first aimed to investigate whether murine SIGNR1 is upregulated in macrophages infected by bacteria.

**Fig. 5:**
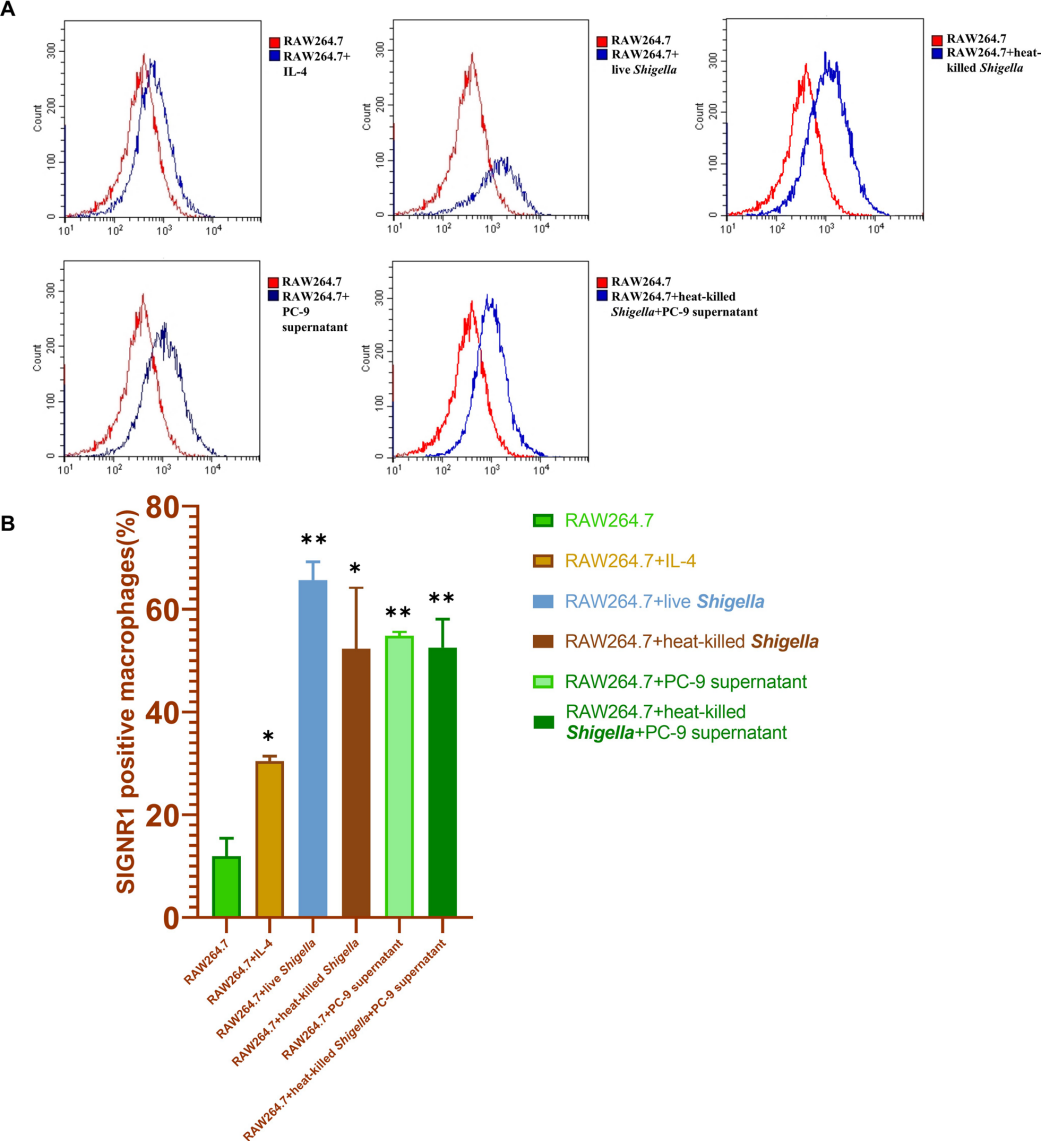
SIGNR1 is upregulated by Gram-negative bacterial infection. **A** RAW264.7 cells were incubated in the presence and absence of lung cancer cell supernatant and were infected with live or heat-killed *Shigella* or treated with IL-4. The level of SIGNR1 expression was then determined using flow cytometry analysis. **B** The quantification of the positive expression of SIGNR1 on RAW264.7 cells (n = 5). The experiment was performed in triplicate. *P < 0.05, **P < 0.005, compared with non-treated RAW 264.7 cells

Flow cytometry data showed that SIGNR1 expression was relatively low in untreated RAW264.7, and that the administration of IL-4 increased its expression (Fig. 5A and 5B). When the RAW264.7 cells were infected with live or heat-killed *S. sonnei*, the number of SIGNR1-positive macrophages became significantly higher than in the control group. Furthermore, the expression of SIGNR1 was the highest on RAW264.7 cells infected with live *S. sonnei*.

Above all, these results indicate that Gram-negative bacterial infection can modify macrophages to express DC-SIGN. This motivated us to further investigate the function of bacteria-modified macrophages in tumor carcinogenesis and metastasis.

### ***S. sonnei***-infected SIGNR1^+^ macrophages can enhance the migration and invasion of lung cancer cells in vitro

**Fig. 6:**
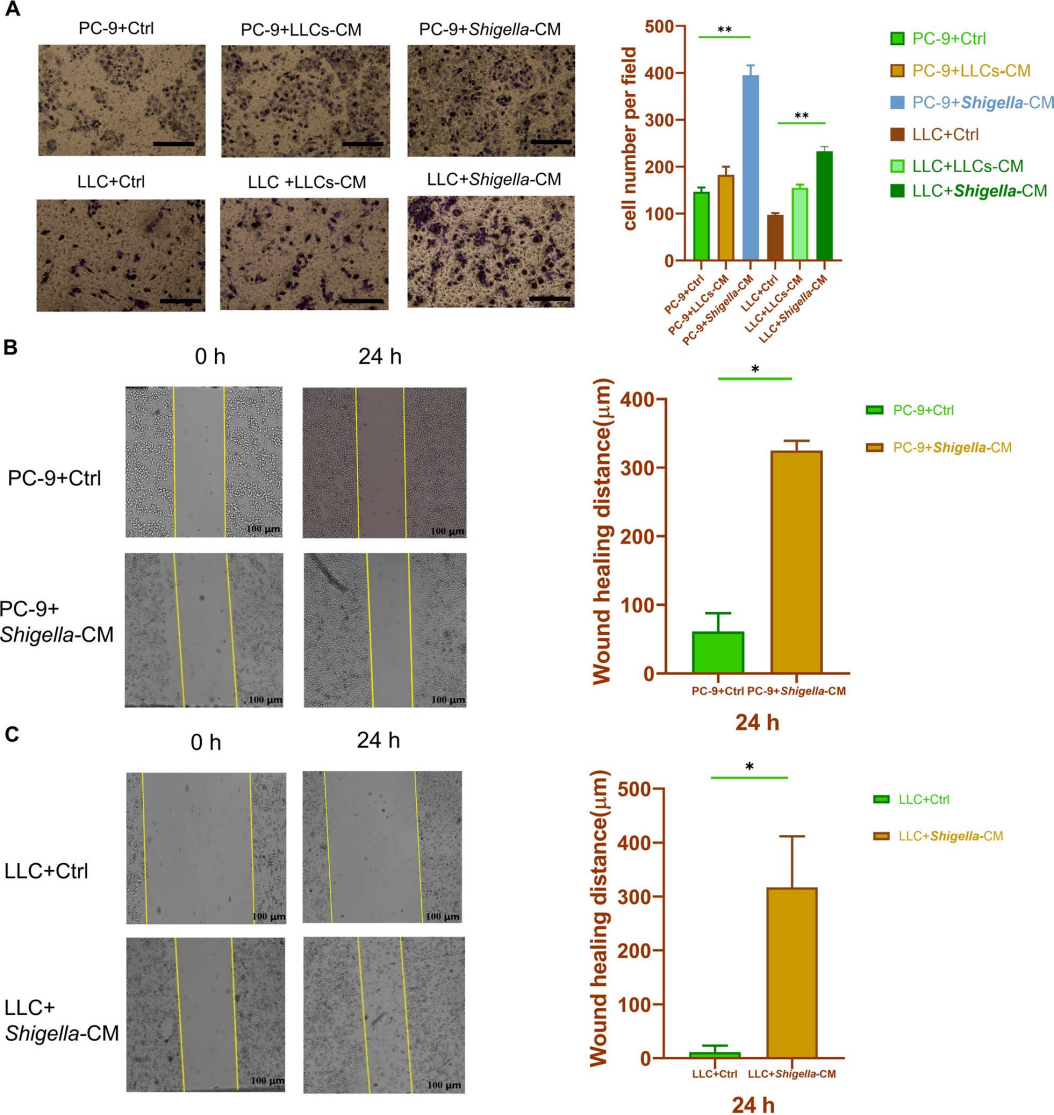
*S. sonnei* bacteria-infected SIGNR1 + macrophage enhances the migration and invasion of lung cancer cells in vitro. **A** Transwell assay. PC-9 and LLC were cultured with LLCS-CM and Shigella-CM, respectively. Scale bar, 200 µm. *P < 0.05, **P < 0.01. (**B**) and **C** Wound-healing assay. PC-9(B) and LLC(C) cells were treated with Shigella-CM, and the cell monolayers were scratched with 200 μL yellow pipette tips. Images were recorded at 0 and 24 h after the wound scratch (left, representative pictures; right, quantitative migrating distance). The yellow lines mark the original wound; the cells moved closer to the wound. The y-axis of the graph is the distance the cells moved. **P < 0.005. LLCS-CM: the conditioned medium of LLC supernatant-treated RAW264.7 cells; Shigella-CM: the conditioned medium of Shigella-infected RAW264.7 cells

Subsequently, we examined whether Gram-negative bacteria-infected SIGNR1^+^ macrophages could enhance PC-9 and LLC cell migration. To address this question, Transwell and wound-healing assays were performed. In the Transwell assays, we used the CM of LLC supernatant-treated RAW264.7 cells (LLCS-CM) and the CM of *Shigella*-infected RAW264.7 cells (*Shigella*-CM) for culture with PC-9 cells and LLC cells. The results showed that PC-9 and LLC cells cultured with *Shigella*-CM showed higher cell migration ability than when cultured with LLCS-CM, or were left untreated (Fig. 6A). Additionally, PC-9 cells exhibited significantly higher migration ability than LLC cells under the same treatment conditions.

We then examined the effect of *Shigella*-CM on lung cancer cell mobility using the wound-healing assay. In the two groups of lung cancer cells; PC-9 and LLC, *Shigella*-CM increased the mobility of the lung cancer cells compared with the controls (Fig. 6C), which exhibited no changes after 24 h.

These results show that *S. sonnei* bacteria-infected SIGNR1^+^ macrophages can promote the progression of lung cancer, as demonstrated by the changes in the mobility of PC-9 and LLC cells. This finding further supports our hypothesis that bacteria-infected macrophages upregulate the expression of SIGNR1, which can interact with lung cancer cells and play a critical role in promoting lung cancer metastasis.

### *S. sonnei* bacteria promotes lung tumor progression and metastasis in murine models

**Fig. 7:**
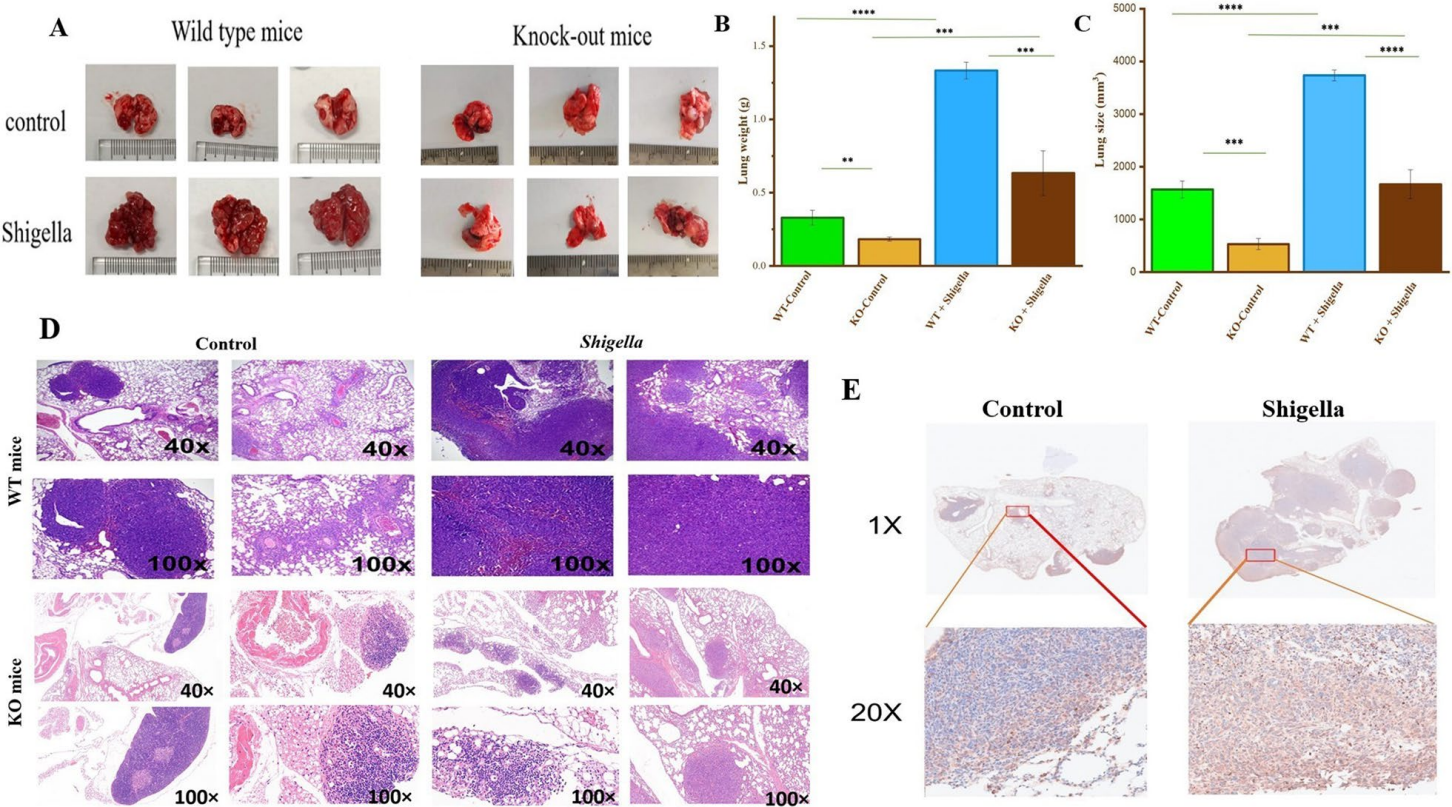
*S. sonnei* bacteria can promote the invasion and metastasis of lung cancer cells in vivo. **A** All the mice were sacrificed, and the lungs were collected. Top, wild-type mice. Bottom, *Shigella*-infected mice, showing visible lung metastases. The same scale (mm^3^) was applied to both groups to eliminate size discrepancies. **B** Quantitative summary of the lung weights (n = 10 mice per group). ****P < 0.0001, *** *P* < 0.005, ** *P* < 0.01. **C** Quantitative summary of the lung cancer sizes (n = 10 mice per group). ****P < 0.0001, *** *P* < 0.005 **D** Hematoxylin and eosin (H&E) staining in the lung metastatic site of the LLC cells. Top, 40 × magnification. Bottom, 100 × magnification. **E** Immunohistochemical analysis of 20 WT murine lung cancers stained with anti-SIGNR1 antibodies. Representative images of cancer tissues show the expression level of SIGNR1 in the two groups (left, n = 10; right, n = 10)

WT and KO mice were separated into two groups, each consisting of 20 mice. In both the WT and KO groups, 10 mice were intranasally inoculated with *S. sonnei* to examine the effect of bacterial infection on lung cancer development. The results are shown in Fig. 7.

The results demonstrated that *Shigella* infection significantly promoted lung tumor growth in WT mice compared to the WT control and KO mice groups (Fig. 7A, 7B, and 7C). Both lung weight and size were significantly higher in the WT mice bacteria-infected group than in the WT control and KO mice groups. In contrast, KO mice infected with *S. sonnei* showed an increase in lung weight and tumor size compared to their uninfected counterparts. However, the extent of this increase was significantly less pronounced than that observed in the *S. sonnei* infected-WT group. This suggests that the DC-SIGN receptor is critical in mediating the response to *S. sonnei* infection and its effect on lung cancer progression. In addition, the results demonstrated that the absence of SIGNR1 decreased lung weight and tumor size in the KO mice control group compared to the WT control group. These results indicated that SIGNR1 promotes lung tumor growth even in the absence of bacterial infections. However, bacterial infection seems to exacerbate lung cancer progression in mice through mechanisms involving the DC-SIGN receptor.

Then, histological analyses were performed on lung tissues harvested from WT and KO mice to evaluate whether *S. sonnei* bacteria promote the development of metastatic lung cancer nodules via DC-SIGN-mediated mechanisms. The results demonstrated significant differences in lung cancer development between WT and KO mice following *Shigella* infection (Fig. 7D). In WT mice, significant alterations in lung histology were observed following *Shigella* infection compared to the uninfected WT control group. In the lung cancer sections from the infected-WT group, there was a significant increase in the infiltration of immune cells, as well as a greater number of metastatic lung cancer nodules. These nodules were more prominent and abundant compared to those in the control group, indicating that *Shigella* infection significantly accelerates metastatic progression in WT mice. In the KO mice, both control and *Shigella*-infected groups exhibited a less dense lung tissue architecture than their WT counterparts (Fig. 7D). The control KO mice exhibited typical lung morphology with few detectable metastatic nodule formations, suggesting that the knockout of SIGNR1 may provide resistance against metastasis progression, as metastatic cancer cells do not seem to accumulate in the lung tissue of KO mice. Following *Shigella* infection, KO mice lung tissues showed fewer metastatic nodules than in the infected WT group. Despite the infection, the lung sections of KO mice did not exhibit the same extent of immune cell infiltration or nodule development observed in the WT mice. This suggested that the absence of the SIGNR1 receptor may inhibit metastatic progression even in the presence of bacterial infection. While the differences in the promotion of metastatic progression were clear in WT mice before and after infection, without the SIGNR1, *Shigella i*nfection does not seem to cause the same level of promotion of nodule formation in KO mice. These results suggested that *S. sonnei* infection promotes metastatic lung cancer progression via DC-SIGN-mediated mechanisms.

To further investigate the effect of *S. sonnei* infection on SIGNR1 expression in vivo, lung tissue specimens from WT murine lung cancer models were analyzed. Tissue samples were stained using an anti-SIGNR1 antibody to detect SIGNR1 expression. Immunohistochemical analysis revealed a significant increase in SIGNR1 expression in the *Shigella*-infected group compared to the control group, as shown in Fig. 7E. These results suggest that Gram-negative bacteria infections such as *S. sonnei* can upregulate SIGNR1 expression in lung cancer tissues to enhance the interaction between lung cancer cells and bacteria, thereby promoting the invasion and metastasis of lung cancer.

Overall, the results of this study indicate that *S. sonnei* infection promotes lung cancer cell invasion and metastasis by increasing SIGNR1 expression, highlighting SIGNR1 as a key mediator in this process. This suggests a novel mechanism by which Gram-negative bacterial infections can exacerbate cancer progression. The interaction between lung cancer cells and Gram-negative bacteria, facilitated by SIGNR1, could provide a new avenue for therapeutic intervention. By targeting the SIGNR1 pathway, it may be possible to disrupt the cancer-promoting effects of bacterial infections, potentially reducing cancer invasiveness and improving patient outcomes.

## Discussion

An increasing number of studies highlight the human microbiome's significant role in the pathophysiology of various human diseases, including cancer. Microbial involvement has been identified in crucial aspects of cancer, such as tumorigenesis, progression, and disease outcome. It has been suggested that around 20% of human cancers could potentially be attributed to microbial influences [107–109], drawing considerable attention to the relationship between microbes and cancer among researchers. Microorganisms within tumors have been recognized for over a century, with numerous studies reporting their existence in various tumor types [110–112]. Notable examples include *Helicobacter pylori*, a member of Proteobacteria, which has been implicated in the development of gastric cancer [113, 114], and *Salmonella Typhi*, associated with the development of gallbladder cancer [115, 116]. In the context of lung cancer, bacterial infections have been identified as a risk factor [18, 117–120]. These infections have been reported as the leading cause of increased mortality in lung cancer patients [121–124]. However, it remains unclear whether infections act as driving factors for lung cancer or merely as bystanders in its development, given that many patients experience lung infections during different stages of cancer progression.

This study first aimed to assess the microbial diversity in lung cancer tissues to understand the microbial landscape associated with lung cancer. To achieve this, 16S rRNA gene sequencing was performed on DNA extracted from lung cancer tissue samples. The results revealed that Proteobacteria was the predominant phylum in lung cancer and adjacent tissues, consistent with previous findings [125–127]. Greathouse conducted a significant study, analyzing 143 lung cancer tissues, and reported Proteobacteria as the primary phylum, with a relative abundance of 70% [127]. Similarly, another study examining 165 adjacent normal lung cancer tissue samples indicated that Proteobacteria was the main phylum, with a relative abundance of 60% [128]. In contrast, Liu et al. reported a significantly lower presence of the Proteobacteria phylum, specifically the genera *Acinetobacter* and *Acidovorax*, in lung cancer tissues of patients with or without emphysema compared to those with emphysema alone [26]. However, they also found that the Proteobacteria member *Escherichia/Shigella* was more prevalent in lung cancer patients than in those with emphysema only [26]. Previous studies have detected *Shigella* species in lung tissues of pneumonia and lung cancer patients [83, 85, 129]. In addition, Nejman et al. analyzed 1,010 tumor samples and 516 controls and found that the Proteobacteria phylum was enriched in the lung tumor samples [32]. Moreover, Morris et al. observed the enrichment of Proteobacteria, specifically the genus of *Enterobacteriaceae*, in lung tissues compared to surrounding sites [130].

Given that Proteobacteria is a major phylum of Gram-negative bacteria [131–133], our findings provide evidence of the coexistence of Gram-negative bacteria within lung cancer tissues.

C-type lectin receptors, such as DC-SIGN, are transmembrane proteins expressed on APCs, including dendritic cells and macrophages [134, 135]. While the role of C-type lectin receptors in pathogen recognition is well-established, their involvement in cancer development remains incompletely understood. Expression of C-type lectin receptors has been reported in various types of tumors [39, 100]. As key players in the immune response, C-type lectin receptors participate in different aspects of anti-tumor immunity. They can recognize glycosylated tumor-associated antigens, thereby inducing an active T-cell response against these antigens [136]. Conversely, tumor cells can exploit C-type lectin receptors to evade immune surveillance [137–140]. The expression of DC-SIGN has been detected in various types of cancer tissues, particularly in patients with metastases [99, 141, 142]. Inhibition of DC-SIGN has been shown to reduce cancer cell invasion, migration, proliferation, and angiogenesis, underscoring its critical role in promoting cancer metastasis [143–147]. Notably, DC-SIGN^+^ cells have been detected in colon cancer tissues in both normal colon mucosa and cancerous tissues [99, 148]. While DC-SIGN staining is weak in normal colon tissues, high expression is observed in colon cancer tissues [99]. Dominguez-Soto et al. reported DC-SIGN expression in stromal cells from gastric adenocarcinoma, breast carcinoma, and pancreatic ductal gastric carcinoma, with colocalization of DC-SIGN and the macrophage marker CD68 [141]. DC-SIGN expressed in tumor stroma cells can induce its expression in peripheral blood monocytes [141].

Consistent with previous findings, this study revealed high levels of DC-SIGN expression in lung cancer tissues compared to adjacent and normal lung tissues, as observed through immunohistochemistry. This finding suggests that DC-SIGN may play a significant role in cancer progression. Zhang et al. also reported DC-SIGN expression in non-small cell lung cancer tissues, with its expression level positively correlated with the metastasis stage of the tumor [47]. While DC-SIGN is primarily found on immature dendritic cells, it has been reported to be expressed on alveolar macrophages in the lung [149]. Therefore, it is unsurprising that our study observed certain levels of human DC-SIGN expression in the remote normal lung tissues, as shown in Fig. 2.

After profiling the bacterial composition in lung cancer tissues and detecting DC-SIGN expression, an adhesion assay was conducted to determine whether DC-SIGN mediates interactions between lung cancer tissues and Gram-negative bacteria. The results revealed that lung cancer tissues expressing DC-SIGN can bind to the LPS core of Gram-negative bacteria, such as *Proteus mirabilis, Shigella sonnei,* and *Yersinia pseudotuberculosis*. The expression of O-antigen in smooth strains of these bacteria weakened this interaction, consistent with previous studies reporting that Gram-negative bacteria can use their LPS core to bind to the DC-SIGN receptor on APCs [61, 70, 71]. An inhibition assay was conducted to confirm the specificity of the interaction involving Gram-negative bacteria, specifically *Shigella sonnei*, which will be utilized in subsequent experiments. This assay aimed to determine whether the interaction between *Shigella sonnei* and lung cancer tissues could be inhibited by anti-hDC-SIGN antibodies and mannan. A significant reduction in this interaction was observed with these interventions, consistent with previous findings [57, 58, 60, 66, 70, 71]. All these results suggested that Gram-negative bacteria may contribute to lung cancer progression by DC-SIGN-mediated mechanisms.

Previous studies have demonstrated that circulating macrophages can migrate to remote tissues in response to inflammatory signals [150–152]. Building on the findings of Wu et al., which demonstrated that rough strains of *S. sonnei* interact with SIGNR1 on macrophages to promote host dissemination [70], we hypothesize that SIGNR1^+^ macrophages may bind to *S. sonnei* in the gut and transport these bacteria to lung tissues, thereby contributing to cancer progression. Beyond their indirect role in cancer initiation and progression, bacteria can proliferate within macrophages and manipulate their functions through various interference mechanisms [153]. This complex interaction between bacteria and macrophages is crucial for advancing our understanding of cancer development. One key aspect of this interaction is macrophage polarization, serving as an intermediary process during tumorigenesis and regression. Macrophages can exhibit different phenotypes, such as M1 (classically activated) or M2 (alternatively activated) macrophages, depending on the signals they receive. Among these phenotypes, M2 macrophages are recognized as TAMs [154–156]. M2-polarized TAMs have consistently demonstrated adverse effects in malignant cancers [155–157]. TAMs are essential components of the tumor microenvironment and play diverse roles in carcinogenesis, neurogenesis, neoangiogenesis, remodeling of the immunosuppressive tumor microenvironment, chemoresistance, recurrence, immune evasion, and metastasis [158–160]. Within the tumor microenvironment, M2 cytokines, including IL-4, IL-13, and IL-10, are present, and TAMs in various cancer models exhibit an M2 activation profile characterized by increased expression of CD163, MRC-1, C-type lectins, IL-10, and Arg-1, as well as reduced production of IL-12 [161–166]. Certain Gram-negative bacteria have been shown to induce M2 polarization of macrophages within the tumor microenvironment, thereby promoting cancer development [153, 167–173]. However, the mechanism by which bacteria within tumors interact with macrophages to promote lung cancer development is not fully understood. DC-SIGN has been identified as a marker of tumor-promoting M2 macrophages [39]. In addition, the presence of DC-SIGN^+^ macrophages in tumor tissues has been associated with tumor development and immune evasion by cancer cells [39]. M2-polarized macrophages expressing DC-SIGN have also been found to enhance the migration of cancer cells [39]. Studies have reported that DC-SIGN^+^ macrophages in the tumor stroma are frequently associated with high levels of vascular endothelial growth factor (VEGF), a key molecule involved in angiogenesis [174]. This suggests that DC-SIGN^+^ macrophages contribute to developing new blood vessels, thereby supporting tumor growth and progression. According to Yan et al. (2016), DC-SIGN^+^ macrophages increased the migration ability of LLCs after 24 h of incubation. In contrast, inhibiting SIGNR1 reduced the percentage of migrated cells compared to the control group [39]. The same study further proposed that murine SIGNR1 expressed in macrophages induced by LLCs contributes to the evasion of lung cancer. In this study using live and heat-killed *S. sonnei* infections, the potential role of Gram-negative bacterial infections and DC-SIGN receptors in modulating macrophage behavior and influencing lung cancer metastasis was highlighted. The results demonstrated that *S. sonnei* infections induce the upregulation of SIGNR1 expression in the murine macrophage cell line RAW264.7, which enhances the migration and invasion ability of lung cancer cell lines in vitro. Combined with previous findings, these results suggest that Gram-negative bacteria can interact with lung cancer tissues via DC-SIGN while simultaneously increasing the expression of this receptor on macrophages, thereby enhancing the interaction between macrophages and lung cancer tissues. This indicates that the DC-SIGN receptor is a key mediator in the interaction between bacteria, macrophages, and lung cancer tissues, promoting lung cancer metastasis in vitro.

Microorganisms can reach lung tissue from the upper airways through migration or from the gastrointestinal tract, where bacteria can enter the circulation through various routes. For instance, intestinal pathogens can travel to the lungs via aspiration of vomit or esophageal reflux. Moreover, disruption of epithelial integrity in the inflamed intestine may facilitate bacterial entry into the circulation, leading to systemic inflammation [175]. The gut-lung axis establishes a link between gastrointestinal diseases and lung diseases [176–180]. Various pathogens, including HIV, Gram-negative bacteria, and parasites, have been found to interact with DC-SIGN on APCs, such as macrophages, to disseminate or persist in the host [181, 182]. This suggests that *S. sonnei* can reach the lungs either via macrophages or through other routes described above to contribute to cancer progression. In this study, the rough strain of *S. sonnei* was nasally administered to mice to simulate infection and investigate its impact on cancer progression once it reached the lungs. This approach was chosen because nasal administration closely mimics how bacteria could enter the respiratory tract through the routes described above. Besides our previous results showing the potential role of the DC-SIGN receptor in mediating the interactions between *S. sonnei*, lung cancer tissues, and macrophages and promoting cancer metastasis in vitro, an in vivo study was performed to examine the role of core LPS-SIGNR1 interaction in promoting lung cancer development and metastasis. To test if the SIGNR1 gene knockout would reduce the contribution of Gram-negative bacterial infection in promoting lung cancer development, SIGNR1 KO mice were employed in this experiment. Several studies have used SIGNR1 KO mice to examine whether the core LPS-SIGNR1 interaction occurs in vivo [62, 63]. The results of this study demonstrated that nasal infection with *S. sonnei* significantly increased the weight and size of lung cancer tumors, as well as the number of metastatic nodules in WT mice compared to KO mice. Furthermore, *S. sonnei* infection significantly increased DC-SIGN expression in lung cancer tissues in wild-type mice. These findings are consistent with in vitro results that suggest that Gram-negative bacterial infections through the DC-SIGN receptor promote lung tumor progression and metastasis. Although several studies have reported the role of Gram-negative bacterial infections in lung cancer development [10, 13, 122, 183–185], to the best of our knowledge, this is the first study to investigate the role of Gram-negative bacterial infection through the DC-SIGN receptor in this specific context.

The results of the present study indicate that Gram-negative bacterial infection, via DC-SIGN-mediated mechanisms, may contribute to lung cancer progression. This suggests that targeting the DC-SIGN receptor presents a promising alternative therapeutic strategy for lung cancer treatment, potentially reducing bacterial infection's role in cancer progression and improving patient outcomes.

## Data Availability

No datasets were generated or analysed during the current study.

## Abbreviations

APCs: Antigen-presenting cells
WT: Wild-type mice
KO: SIGNR1 knockout
NSCLC: Non-small-cell lung cancer
SCLC: Small-cell lung cancer
AC: Adenocarcinoma
SCC: Squamous cell carcinoma
TME: Tumor microenvironment
EMT: Epithelial-mesenchymal transition
MMPs: Matrix metalloproteinases
TAMs: Tumor-associated macrophages
CRDs: Carbohydrate recognition domains
DC-SIGN: Dendritic cell-specific intercellular adhesion molecule-3-grabbing non-integrin
S. sonnei: Shigella sonnei
M cells: Microfold cells
IACUCs: Institutional animal care and use committees
IRB: Institutional review board
LLC: Lewis lung carcinoma
pYV: Virulence plasmid
FBS: Fetal bovine serum
IL-4: Interleukin 4
PBS: Phosphate buffered saline
LB: Luria–Bertani
CFUs: Colony-forming units
PFA: Paraformaldehyde
PCR: Polymerase chain reaction
16S rRNA: 16S ribosomal RNA
PacBio: Pacific biosciences
SMRT: Single-molecule real-time
CM: Conditioned medium
OTU: Operational taxonomic unit
LPS: Lipopolysaccharide
VEGF: Vascular endothelial growth factor
HIV: Human immunodeficiency virus
